# Pharmacometric‐Based Evaluation of Salmeterol and Its Metabolite α‐Hydroxysalmeterol in Plasma and Urine: Practical Implications for Doping Control

**DOI:** 10.1002/psp4.70187

**Published:** 2026-01-16

**Authors:** Paul Thoueille, Anne Danion, Morten Hostrup, Michael Petrou, Koen Deventer, Thierry Buclin, François R. Girardin, Irene Mazzoni, Olivier Rabin, Monia Guidi

**Affiliations:** ^1^ Service and Laboratory of Clinical Pharmacology Lausanne University Hospital and University of Lausanne Lausanne Switzerland; ^2^ Science and Medicine Department World Anti‐Doping Agency Montreal Quebec Canada; ^3^ Department of Nutrition, Exercise and Sports, the August Krogh Section for Human Physiology University of Copenhagen Copenhagen Denmark; ^4^ Cyprus Anti‐Doping Authority Makarion Athletic Centre Avenue Engomi Cyprus; ^5^ Doping Control Laboratory (DoCoLab), Ghent University Department of Diagnostic Sciences (GE32) Ghent Belgium; ^6^ Centre for Research and Innovation in Clinical Pharmaceutical Sciences Lausanne University Hospital and University of Lausanne Lausanne Switzerland; ^7^ Institute of Pharmaceutical Sciences of Western Switzerland University of Geneva, University of Lausanne Lausanne Switzerland

**Keywords:** anti‐doping, NONMEM, population pharmacokinetics, salmeterol, WADA

## Abstract

Salmeterol is a commonly used β_2_‐agonist included on the List of Prohibited Substances and Methods published by the World Anti‐Doping Agency (WADA). We developed a population pharmacokinetic (popPK) model to describe the PK of salmeterol including its major metabolite, α‐hydroxysalmeterol, in plasma and urine after inhalation. The model was used to evaluate the ability of the current minimum reporting level (MRL) of 10 ng/mL for salmeterol to discriminate between permitted and prohibited use of salmeterol. Six studies on healthy participants, chronic asthmatics, or athletes were pooled and provided a total of 1175 concentrations (275 and 398 for salmeterol and 185 and 317 for α‐hydroxysalmeterol in plasma and urine, respectively) from 92 individuals. A two‐compartment model assuming intravenous‐like bolus absorption best depicted plasma salmeterol PK, with a complete parent conversion into α‐hydroxysalmeterol. Because urine volumes were only recorded in two studies, a separate urine compartment was defined to approximate physiologic micturition. Athletes had a 63% higher salmeterol plasma clearance and a 191% greater salmeterol urinary rate constant compared to other subjects, resulting in significantly higher salmeterol urine concentrations. Our popPK model suggests that salmeterol concentrations in urine at therapeutic doses (100 μg twice daily) are unlikely to be reported using the current MRL. However, to improve its sensitivity to detect cases of doping, an adjustment in the MRL and/or a different analytical target would be recommended.

## Study Highlights

- What is the current knowledge on the topic?
  ○ Salmeterol is a commonly used β_2_‐agonist included on the List of Prohibited Substances and Methods of the World Anti‐Doping Agency (WADA) with a specified dosing limit of 200 μg in any 24 h period via inhaled administration. A minimum reporting level of 10 ng/mL is currently used to flag urine doping samples as an indication of doping.
- What question did this study address?
  ○ Our model‐based meta‐analysis was performed to provide the basis for evaluating the ability of the current WADA approach to discriminate between the permitted and prohibited use of salmeterol.
- What does this study add to our knowledge?
  ○ This study provides a comprehensive description of the pharmacokinetics of salmeterol and its major metabolite in plasma and urine and discusses the impact of different dosing regimens on the urine concentrations of salmeterol in athletes/endurance‐trained individuals.
- How might this change drug discovery, development, and/or therapeutics?
  ○ The model‐based simulations suggest that the current approach for evaluating suspected salmeterol misuses could be improved by revising the dosing regulations, refining the analytical methodology, and/or adjusting the MRL.

## 1 Introduction

Inhaled β_2_‐agonists are among the drugs most frequently used in sports, which is related to a high prevalence of asthma and exercise‐induced bronchoconstriction among athletes [1]. Because β_2_‐agonists have the potential to enhance performance when used via systemic routes or at high inhaled doses [2, 3, 4], the World Anti‐Doping Agency (WADA) has imposed restrictions on their use in and out of competition as per the List of Prohibited Substances and Methods (i.e., the Prohibited List) [5]. The β_2_‐agonists commonly used in asthma are allowed at standard inhaled doses, assumed to be devoid of substantial doping effects. This ensures that athletes can be treated without concerns of committing an anti‐doping rule violation, while at the same time lifting the administrative burdens associated with systematic requests for therapeutic use exemptions. On the other hand, doping controls are performed to detect cases of overexposure and limit the potential for misuse. For other exempted inhaled β_2_‐agonists, namely salbutamol and formoterol, WADA bases the detection of misuse on urinary thresholds and corresponding decision limits in doping control samples. Thus, the finding of a urine concentration exceeding 1000 ng/mL of salbutamol or 40 ng/mL of formoterol is considered inconsistent with a standard therapeutic dosage of the substance. However, there is currently no such threshold or decision limit for salmeterol.

Salmeterol is a long‐acting β_2_‐agonist [6, 7] used in the treatment of unstable asthma, typically in combination with an inhaled glucocorticoid [8]. It is commonly prescribed for twice‐daily inhalation at doses ranging from 25 to 100 μg, and the Prohibited List specifies an upper dosing limit of 200 μg in any 24 h period [5]. In the absence of a defined urinary threshold, WADA‐accredited laboratories practically apply a minimum reporting level (MRL) of 10 ng/mL to distinguish a urinary concentration compatible with a permitted therapeutic use from a nontherapeutic prohibited use [9]. If a urine doping control sample exceeds the MRL, this is flagged as an Adverse Analytical Finding (AAF), which typically warrants further inquiry. Nevertheless, since the introduction of this MRL for salmeterol, no AAFs have been reported. This likely means that most athletes who use salmeterol adhere to standard prescribed doses and confirms that the current MRL does not put athletes at risk of being unduly penalized. However, the current MRL definition is based only on limited data regarding the pharmacokinetics (PK) of salmeterol, which is in part due to fairly low urinary concentrations observed at therapeutic doses, difficult to measure with the analytical methods available at the time of salmeterol commercialization [7].

Given that more data are now available, it would be beneficial to fine‐tune the MRL to ensure sufficient sensitivity to detect misuse. The question is whether the highest salmeterol concentrations quantified below the MRL are actually compatible with a permitted use. In addition, metabolites are increasingly being used as ancillary indicators to distinguish doping from legitimate use [10]. Because salmeterol undergoes extensive cytochrome P450 3A4‐mediated hydroxylation into its major metabolite α‐hydroxysalmeterol, parent salmeterol only accounts for less than 5% of the dose excreted in urine [6]. Thus, for refining the salmeterol MRL, it could also be relevant to assess the potential utility of α‐hydroxysalmeterol as a doping control marker [11, 12].

This study aimed to provide the basis for evaluating the ability of the current MRL to discriminate between the permitted and prohibited use of salmeterol. To this end, we developed a population pharmacokinetic (popPK) model to describe the PK of salmeterol, including its major metabolite α‐hydroxysalmeterol, in both plasma and urine after salmeterol inhalation.

## 2 Methods

### 2.1 Data

Six published and one unpublished study supported by WADA were aggregated for the popPK analysis [11, 12, 13, 14, 15, 16]. The studies were identified by the WADA scientific staff and through a literature search in PubMed, using the following query: “salmeterol AND (concentration* OR pharmacokinetic*) AND urine AND human*” (Figure S1). Table 1 summarizes the study characteristics relevant for the model development. It should be noted that the information reported was heterogeneous, for example, regarding quantification methods [i.e., analytical methods, lower limits of quantification (LLOQ)] and type of data (i.e., availability of urine specific gravity [USG, see below), volume of urine collections and quantified compounds]. More specifically, Jacobson et al. [14] quantified the R‐ and S‐salmeterol enantiomers, whose concentrations were summed up to be consistent with the other studies. For the same reason, we retained the concentrations measured using enzymatic hydrolysis for sample preparation from the study by Deventer et al. [16]. To account for the hydration status of study subjects at the time of urine collection, urine concentrations were corrected for USG according to WADA technical documents [17], when USG was available:

$$\begin{array}{c} \begin{array}{c} \mathrm{USG}-\text{corrected concentration}=\frac{1.020-1}{\left(\mathrm{USG}+0.002\right)-1}\times \text{observed concentration} \end{array} \end{array}$$

where 1.020 is the specific gravity threshold for the USG correction, USG refers to the actual USG of the sample with 0.002 added to account for the uncertainty in the specific gravity measurement.

**TABLE 1:** Relevant characteristics for the model development.

Study	*n*	Type of individuals	Device	Study plan	Concentrations available	Urine volume	USG
Jacobson et al. [12]	7 14	Healthy Athletes	DPI	100 μg SD 200 μg SD	52 urine salmeterol 57 urine α‐hydroxysalmeterol	—	Available
Jessen et al. [11]	11	Endurance‐trained	DPI	Trial 1: 400 μg SD Trial 2: 1 week washout + 200 μg SD Trial 3: 200 μg 7‐day self‐administration	179 plasma and 106 urine salmeterol 185 plasma and 106 urine α‐hydroxysalmeterol	Available	Available
Bozzolino et al. [13]^a^	10	Asthmatics	MDI	50 and 100 μg doses at steady‐state	10 urine salmeterol	—	—
Jacobson et al. [14]	10	Healthy	MDI DPI	50 μg MDI (*n* = 6) SD 200 μg DPI (*n* = 4) SD	39 urine salmeterol	—	—
Hostrup et al. [15]	10 10	Healthy Asthmatics	DPI	100 μg SD	60 plasma and 60 urine salmeterol 60 urine α‐hydroxysalmeterol	—	Available
Deventer et al. [16]	6	Healthy	MDI	100 μg SD	37 urine salmeterol	Available	—
Petrou et al. (unpublished)	24	Healthy	DPI	Trial 1: 100 μg SD Trial 2: 1 week washout + 100 μg SD	36 plasma and 104 urine salmeterol 94 urine α‐hydroxysalmeterol	—	Available

Abbreviations: DPI, dry powder inhaler; MDI, metered dose inhaler; *n*, number of individuals; SD, single dose; USG, urine specific gravity.

^a^ Removed from the analysis (see explanations in Methods).

**FIGURE 1:**
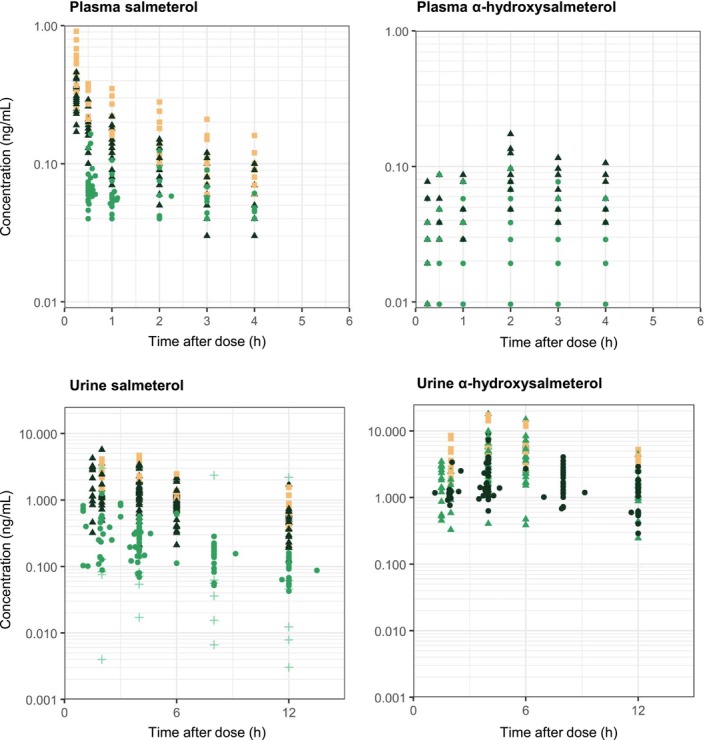
Observed concentrations versus time after dose for salmeterol and α‐hydroxysalmeterol in both plasma (upper panels) and urine (lower panels). Data were retrieved from the six studies sponsored by the WADA that fulfilled the study inclusion criteria. Cross: 50 μg; Dot: 100 μg; Triangle: 200 μg; Square: 400 μg. Color code according to study.

Finally, concentrations with unreliable information on the time of last dose administration, blood sampling, or urine collection were removed from the analysis. Therefore, the data collected in patients under chronic therapy from the study of Bozzolino et al. [13] had to be excluded. Figure 1 shows the observed concentrations of salmeterol and its metabolite as a function of time after dose in both plasma and urine.

### 2.2 Population Pharmacokinetics Analysis

We conducted the popPK analysis using the non‐linear mixed effects modeling software NONMEM (v7.5.1) assisted by PsN (v5.3.1) and Pirana (v2.9.3). We performed the data management and graphical and statistical analyses with R (v4.1.1). Artificial Intelligence was used to improve readability and language of the manuscript.

Salmeterol doses were converted to nmol, and the measured concentrations of salmeterol and its metabolite α‐hydroxysalmeterol to nmol/L. The M1 (i.e., discarding data below the quantification limits (BQL)), M3 (i.e., likelihood‐based method with Laplacian option), and M6 (i.e., replacing the first BQL observation with LLOQ/2 of the study analytical method and discarding subsequent BQL values) approaches were evaluated for handling BQL data [18].

**FIGURE 2:**
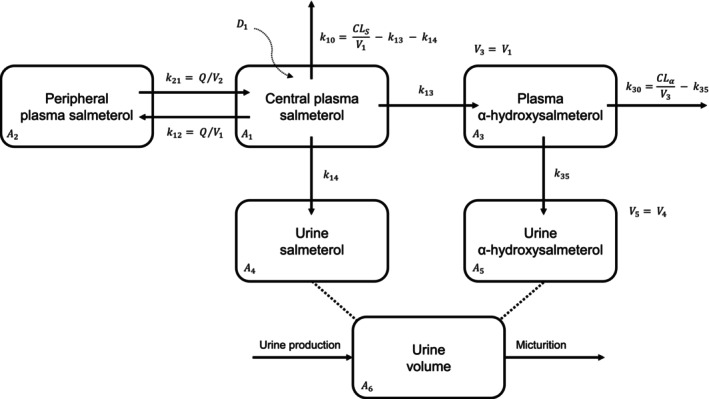
Structural model used to describe salmeterol concentration‐time profiles. A_x_, drug amount in the corresponding compartment, CL_S_/F, apparent clearance of plasma salmeterol, CL_α_/F, apparent clearance of plasma α‐hydroxysalmeterol, D_1_, instantaneous injection, k_10_, elimination rate constant for plasma salmeterol, k_12_, rate transfer constant of central to peripheral plasma salmeterol, k_13_, rate constant from plasma salmeterol to α‐hydroxysalmeterol, k_14_, urinary excretion rate constant of salmeterol, k_21_, rate transfer constant of peripheral to central plasma salmeterol, k_30_, elimination rate constant for plasma α‐hydroxysalmeterol, k_35_, urinary excretion rate constant of α‐hydroxysalmeterol, Q/F, apparent intercompartmental clearance, V_1_/F, apparent central volume of plasma salmeterol, V_2_/F, apparent peripheral volume of plasma salmeterol, V_3_/F, apparent volume of plasma α‐hydroxysalmeterol, V_4_/F, urine volume of salmeterol, V_5_/F, urine volume of α‐hydroxysalmeterol.

Figure 2 shows the structural model developed for the description of salmeterol and α‐hydroxysalmeterol in plasma and urine. The model was implemented as a system of differential equations (ADVAN13 subroutine in NONMEM). Initially, we developed a popPK model for salmeterol plasma concentrations collected in two published studies [11, 15] and the unpublished study by Petrou et al. (Table 1), and then we included the α‐hydroxysalmeterol plasma data from Jessen et al. [11]. We subsequently included salmeterol (all studies) and α‐hydroxysalmeterol (four studies [11, 12, 15], including the Petrou study) urine concentrations. Data formatting is detailed in Data S1.

#### 2.2.1 Base and Covariate Models

Because salmeterol is deposited in the lungs and also swallowed and ingested [6, 19], we first tested a parallel absorption process with a fraction of the dose, constrained between 0 and 1 using a logit equation [20], released via a relatively fast absorption pathway. However, due to the rapid pulmonary absorption of salmeterol, with maximum concentrations obtained in less than 20 min [6], the analysis indicated extremely fast absorption pathways. Therefore, intravenous‐like bolus absorption kinetics was found more appropriate, in accordance with previous observations [21]. One‐ and two‐compartment models with linear elimination for plasma salmeterol were compared. Then, a complete and irreversible plasma conversion of salmeterol into α‐hydroxysalmeterol (k_13_), with linear elimination of the latter, was assumed to describe plasma α‐hydroxysalmeterol concentrations [6]. Note that PK parameters estimates should be interpreted as apparent values in the absence of intravenous data. The volumes for the plasma compartments (i.e., V_1_/F and V_3_/F) were assumed to be equal to handle identifiability issues. Urine salmeterol and its metabolite were then modeled using two distinct compartments connected to their respective plasma compartments through the constant rates k_14_ and k_35_. When available (i.e., two studies [11, 16]), urine volumes (i.e., V_4_/F and V_5_/F) were directly used. For all the other studies, a separate urine compartment was defined to approximate physiologic micturition, assuming constant urine production per hour (UR_PROD) to derive urine volumes (V_6_/F) (See Data S1). Urine concentrations were fitted by dividing drug/metabolite amounts by the volume produced in the corresponding period [22].

All parameters were assumed to follow log‐normal distributions. Inter‐individual variability (IIV) was sequentially tested on all parameters, including UR_PROD with and without the USG correction. Mixed, proportional, and additive error models with and without correlation (i.e., L2 item in NONMEM) specific to the study and matrices were compared to evaluate the residual unexplained variabilities (RUV) of salmeterol and its metabolite in plasma and urine. RUVs were regrouped when deemed similar for the same matrices and the same compound. Multiple levels of random effects (i.e., individual and study‐level IIV) [23] were also tested on all parameters.

Due to the limited availability of covariates, we were only able to evaluate two categorical covariates: the type of individuals (i.e., classified as healthy participants, chronic asthmatics, and healthy endurance‐trained individuals or athletes) and the administration device (metered dose inhaler or dry powder inhaler). Individual type was first modeled, assigning a separate PK parameter per group (rich model), and then per regrouped type (reduced model). All participants were assumed to have normal renal function.

#### 2.2.2 Model Selection and Evaluation

The difference in the NONMEM objective function value (ΔOFV) served as the main metric for discriminating hierarchical models during the forward model building phase, with a significance level of 0.05 (ΔOFV < −3.84) for the addition of one parameter. In the subsequent backward deletion step, a significance level of 0.01 was employed (ΔOFV > 6.63) for the removal of one parameter. Non‐nested models were discriminated using the Akaike information criterion. Model selection was also guided by diagnostic plots and the precision of PK parameter estimates, as measured by the relative standard error (RSE). In addition, the findings were evaluated by examining model shrinkage and the normality of the distribution of individual eta estimates (reflecting IIV) and of residuals (reflecting RUV).

The 5th, 50th, and 95th prediction and observed percentiles were compared using prediction‐corrected visual predictive checks (pcVPCs) of the final popPK model [24, 25, 26]. Finally, the original model estimates were evaluated against the median parameter values obtained through bootstrapping, along with their respective 95% confidence intervals derived from 2000 replicates [24].

#### 2.2.3 Model‐Based Monte Carlo Simulations

Model‐based Monte Carlo simulations accounting for both IIV and RUV were performed to generate predicted PK profiles for salmeterol and its metabolite α‐hydroxysalmeterol in plasma and urine according to the retained covariates by simulating 1000 individuals in each group. Bladder voiding was assumed to occur every 4 h and prior to salmeterol inhalation, and physiological urine volumes were simulated using the constant urine production (i.e., UR_PROD). This means that each point of the simulated urine PK profile represents the concentration that would be obtained if a urine sample were collected at that moment (e.g., small volumes of urine immediately after inhalation resulting from the constant production of urine after voiding the bladder). To explore the ability of the current MRL to discriminate between the permitted and prohibited use of salmeterol, we simulated different dosing regimens administered over 1 week (i.e., maximum doses authorized [5]) 100 μg at 8 h and 16 h, 100 μg every 12 h (8 h and 20 h), or 200 μg once daily (8 h); prohibited doses: 200 μg at 8 h and 16 h, 200 μg every 12 h (8 h and 20 h). A 99.9th prediction percentile was calculated on 10,000 virtual individuals to explore the “worst‐case scenario”, representing the highest expected salmeterol and α‐hydroxysalmeterol urine concentration under the given dosing conditions. This threshold may ensure that the risk of false positives remains statistically negligible, given that a positive doping test result could have career‐ending consequences for athletes.

## 3 Results

A total of 1175 concentrations (275 and 398 for salmeterol and 185 and 317 for α‐hydroxysalmeterol in plasma and in urine, respectively) measured in 92 individuals were available (see Table 1 and Figure 1). There was a median of 6 (range: 1–58) observations per individual, and 209 levels were below LLOQ. The M1 method was found best suited to handle BQL data (see Model evaluation section below). Therefore, 966 concentrations (258 and 289 for salmeterol and 185 and 234 for α‐hydroxysalmeterol in plasma and urine, respectively) measured in 85 individuals were analyzed. Note that the USG correction could only be performed on 42% and 50% of the salmeterol (120 levels) and α‐hydroxysalmeterol (118 levels) urine concentrations, respectively.

### 3.1 Structural, Statistical, and Covariate Models

#### 3.1.1 Plasma Concentrations

The absorption of salmeterol in plasma was modeled as an intravenous bolus. In accordance with previous observations [21], a two‐compartment model (i.e., $V_{1}/F$ and $V_{2}/F$ for the central and peripheral compartments, respectively) provided the best description of the plasma salmeterol PK (ΔOFV = −127, *p* < 0.0001, compared to a one‐compartment model). IIV was supported for each parameter (ΔOFV < −37, *p* < 0.001) and a common proportional error model for all studies best depicted plasma salmeterol RUV. Then, the plasma conversion of salmeterol into α‐hydroxysalmeterol was included (k_13_) [6]. IIV on k_13_ was retained (ΔOFV = −84, *p* < 0.0001), and α‐hydroxysalmeterol RUV was best described by a proportional model. A 53% correlation between the RUVs of salmeterol and its metabolite was identified for the study by Jessen et al. [11] (ΔOFV = −41, *p* < 0.0001).

Covariate analyses revealed that chronic asthmatics had the lowest salmeterol clearance (CL_S_/F), but not significantly different from healthy participants. These populations were regrouped, and the reduced model showed that athletes/endurance‐trained individuals had a 63% higher CL_S_/F compared to other subjects (ΔOFV = −14, *p* < 0.001), thus resulting in lower salmeterol plasma concentrations. No significant effect of the administration device was found (ΔOFV > −1, *p* > 0.05).

#### 3.1.2 Urine Concentrations

Two distinct compartments were then added to the developed plasma model to account for both salmeterol and its metabolite urine data (see 2. Methods and Data S1). IIV was retained on k_14_ and for UR_PROD when concentrations were not corrected by the USG (ΔOFV = −44 and −31, *p* < 0.0001, respectively). Indeed, the correction of USG is used to account for individual hydration status, which in turn reduces the variability observed in urine production. Proportional models regrouped according to their magnitude (i.e., low, medium, and high variability) best described salmeterol and α‐hydroxysalmeterol RUVs in urine (See NONMEM code in Data S1). In addition, a 61% correlation between the RUVs of salmeterol and its metabolite was identified for the study by Jessen et al. [11] (ΔOFV = −39, *p* < 0.0001). Study‐level IIV did not improve data description.

Finally, like for CL_S_/F, chronic asthmatics had the slowest k_14_ values but were not significantly different from healthy participants (during univariate and multivariate analyses). The reduced model showed that athletes/endurance‐trained individuals had a 191% higher k_14_ compared to other subjects (ΔOFV = −55, *p* < 0.0001), resulting in significantly higher salmeterol urine concentrations. Again, no effect of the administration device was identified (ΔOFV > −2, *p* > 0.05).

#### 3.1.3 Handling of BQL Data

During the first step of the analysis, the M1, M3, and M6 methods were compared to handle BQL data [18]. The M3 and M6 methods were found to be insufficiently robust due to limited data reliability. Indeed, the available data for some individuals showed fluctuating concentrations below and above the LLOQ, which made the proper implementation of these methods difficult. The M1 method was employed to handle BQL data.

### 3.2 Model Evaluation

The estimation of individual‐ and study‐level IIVs was not retained, either based on statistical considerations (i.e., non‐significant and/or minimal variability) or because of poor precision estimation (i.e., RSE > 100%). Goodness‐of‐fit diagnostic plots of the final model were satisfactory (see Figure S2). Note that the plasma concentrations of α‐hydroxysalmeterol lack granularity due to the very low concentrations measured (pg/mL range), resulting in a horizontal arrangement of the data along the line of identity. The inclusion of covariates (i.e., individual types on CL_S_/F and k_14_) improved IIV distribution, restoring a normal distribution centered around zero (see Figure S3).

The pcVPC (Figure S4) and the bootstrap results, presented in Table 2 together with the final model parameters, confirm the reliability of the final model. Despite some modest model misspecifications, probably due to the limited availability and heterogeneity of data at specific timepoints and/or for some matrices, the pcVPC supports the adequacy of the model developed. On the other hand, the differences between bootstrap median values and population estimates do not exceed 7% for any of the parameters, demonstrating excellent agreement.

**TABLE 2:** Final population PK parameter estimates with their bootstrap evaluations.

Parameters	Final model	Bootstrap (*n* = 2000)
	Estimate (RSE, %)	Median [95% CI]
$V_{1}/F$ **(L)**	**446 (17)**	**446 [356–524]**
$\omega_{V_{1}}$ (CV%)	16 (54)	16 [5–22]
Q/F **(L/h)**	**1490 (22)**	**1443 [1121–2144]**
$\omega_{Q}$ (CV%)	86 (37)	81 [33–174]
$V_{2}/F$ **(L)**	**871 (12)**	**873 [766–985]**
$\omega_{V_{2}}$ (CV%)	43 (29)	43 [28–62]
${\mathrm{CL}}_{S}/F$ **(L/h)**	**193 (10)**	**192 [154–231]**
$\omega_{{\mathrm{CL}}_{S}}$ (CV%)	33 (27)	31 [16–44]
$\theta_{\text{Athletes}}$	1.63 (21)	1.64 [1.23–2.16]
$k_{13}$ (**h** ^**−1**^)	**0.30 (16)**	**0.30 [0.23–0.39]**
$\omega_{k_{13}}$ (CV%)	41 (23)	41 [25–54]
${\mathrm{CL}}_{\alpha }/F$ **(L/h)**	**233 (22)**	**232 [177–293]**
$k_{14}$ (**h** ^**−1**^)	**0.00094 (22)**	**0.00095 [0.00075–0.0012]**
$\omega_{k_{14}}$ (CV%)	29 (41)	27 [19–37]
$\theta_{\text{Athletes}}$	2.91 (15)	2.91 [2.28–3.70]
$k_{35}$ (**h** ^**−1**^)	**0.015 (22)**	**0.015 [0.012–0.017]**
UR_PROD **(L/h)**	**0.079 (18)**	**0.080 [0.062–0.100]**
$\omega_{\mathrm{UR}\_{\text{PROD}}_{\mathrm{USG}-\text{uncorrected}}}$ (CV%)	73 (19)	72 [40–109]
$\sigma_{\text{plasma salmeterol}}$ (CV%)	22 (13)	22 [19–24]
$\sigma_{\text{plasma}\;\alpha -\text{hydroxysalmeterol}}$ (CV%)	38 (13)	38 [31–45]
Correlation $\sigma_{\text{plasma}}$ (%)	53 (26)	52 [33–65]
$\sigma_{\text{urine salmeterol}\;\left(\mathrm{low}\right)}$ (CV%)	29 (19)	29 [22–35]
$\sigma_{\text{urine salmeterol}\;\left(\mathrm{mid}\right)}$ (CV%)	41 (21)	40 [36–46]
$\sigma_{\text{urine}\;\alpha -\text{hydroxysalmeterol}\;\left(\mathrm{mid}\right)}$ (CV%)	38 (15)	38 [31–45]
$\sigma_{\text{urine salmeterol}\;\left(\text{high}\right)}$ (CV%)	57 (26)	57 [41–75]
$\sigma_{\text{urine}\;\alpha -\text{hydroxysalmeterol}\;\left(\text{high}\right)}$ (CV%)	51 (13)	51 [41–61]
Correlation $\sigma_{\text{urine}\;\left(\text{Jessen}\;\mathrm{et}\;\mathrm{al}.\right)}$ [11] (%)	61 (30)	60 [44–73]

*Note:* Coefficient of variation (CV, %) for IIV calculated as follows: $\sqrt{\left(e^{\omega^{2}}-1\right)}$. Coefficient of variation (CV, %) for proportional residual errors calculated as follows: $\sqrt{\omega^{2}}$. Correlation expressed as a percentage calculated as follows: $\text{Correlation}\;\left(\%\right)=\frac{{\text{Covariance}}_{\left(\sigma_{\text{parent}-\text{metabolite}}\right)}}{\sqrt{\left(\sigma_{\text{parent}}^{2}\times {\;\sigma }_{\text{metabolite}}^{2}\right)}}$. Relative standard error (RSE) of ω expressed as a percentage, with standard error (SE), calculated as follows: $\mathrm{RSE}\;\left(\%\right)=\frac{e^{\omega^{2}}\cdot \mathrm{SE}}{2\cdot \left(e^{\omega^{2}}-1\right)}$. Bold values represent the key theta parameters, allowing the reader to quickly identify and focus on the most significant data points within the table.

Abbreviations: CL_S_/F, apparent plasma clearance of salmeterol; CL_α_/F, apparent plasma clearance of α‐hydroxysalmeterol; k_13_, plasma conversion of salmeterol into α‐hydroxysalmeterol; k_14_, urinary excretion rate constant of salmeterol; k_35_, urinary excretion rate constant of α‐hydroxysalmeterol; Q/F, apparent intercompartmental clearance; UR_PROD, urine production per hour; USG, specific gravity of urine; V_1_/F, apparent central volume of distribution for plasma salmeterol with F being the relative bioavailability; V_2_/F, apparent peripheral volume of distribution for plasma salmeterol; θ, athlete/trained individuals effect on CL_S_ and k_14_; σ, proportional residual error; ω, inter‐individual variability (IIV).

### 3.3 Model‐Based Monte Carlo Simulations

**FIGURE 3:**
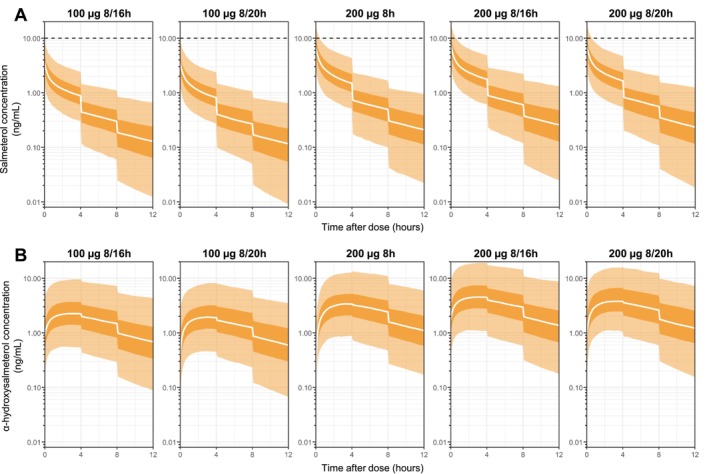
Model‐based simulations of urine concentrations (corrected by the urine specific gravity) following 1 week of administration across different dosing regimens [i.e., maximum doses authorized: 100 μg at 8 and 16 h, 100 μg every 12 h (8 and 20 h), or 200 μg once daily (8 h); prohibited doses: 200 μg at 8 and 16 h, 200 μg every 12 h (8 and 20 h)] in athletes/endurance‐trained individuals (*n* = 1000) with bladder voiding every 4 h. The solid white lines represent the median (50% percentile), while the dark surfaces encompass the 50% prediction intervals, and the light surfaces the 95% prediction intervals. Each point of the simulated urine PK profile represents the concentration that would be obtained if a urine sample were collected at that time point. **(A)** Prediction percentiles of urine concentrations of salmeterol. The horizontal dashed line corresponds to the minimum reporting level of 10 ng/mL. **(B)** Prediction percentiles of urine concentrations of α‐hydroxysalmeterol. For the regimen in which salmeterol is administered twice a day, the simulated concentrations displayed correspond to those following the second dose.

Figure 3 presents the simulated urine concentrations for salmeterol (panel A) and α‐hydroxysalmeterol (panel B), respectively, after different dosing regimens (i.e., authorized and prohibited doses) administered for 1 week in athletes/endurance‐trained individuals. Model‐based simulations show that the median maximum salmeterol urine concentration (which would be the concentration measured directly after the bladder voiding and subsequent drug inhalation) was approximately 3.6 [95% prediction interval (PI_95_): 1.4–9.4] ng/mL and 7.2 [PI_95_: 2.7–19.2] ng/mL under doses of 100 and 200 μg administered twice daily, respectively. Predictions contrasting athletes and asthmatics/healthy participants under the same dosage of 100 μg salmeterol every 12 h are detailed in the Supplement Material (Figure S5).

Additionally, Table 3 summarizes the 2.5%, 50%, 97.5%, 99%, and 99.9% predicted concentrations of salmeterol and its metabolite in urine directly after salmeterol inhalation and at 30 min post‐dose for the different regimens tested in athletes/endurance‐trained individuals and healthy participants. None of the salmeterol urine concentrations measured under the 100 μg regimens are expected to exceed the current MRL of 10 ng/mL under practical conditions (i.e., 30 min post‐dose), regardless of the population considered. Simulations indicated probabilities of 4.1% and 3.3% for salmeterol urine concentrations to exceed the current MRL 30 min after the last dose when 200 μg was administered at 8/16 h and 8/20 h (twice daily), respectively. The probabilities predicted for salmeterol administration of 200 μg once daily and 200 μg twice daily were similar across sampling times, suggesting that no significant drug accumulation is expected when comparing single‐dose and repeated‐dose administration regimens. Finally, Table S1 and Figure 3B showed that the predicted α‐hydroxysalmeterol exhibits a flatter PK profile, with higher urine concentrations around T_max_ (i.e., 2–4 h) compared to salmeterol. This means that α‐hydroxysalmeterol urine concentrations are not as variable over time, making the timing of the sample less critical than for salmeterol quantification.

**TABLE 3:** Predicted urine concentrations for the different salmeterol regimens tested.

			Salmeterol concentration (ng/mL)					α‐Hydroxysalmeterol concentration (ng/mL)				
			2.5%	50%	97.5%	99%	99.9%	2.5%	50%	97.5%	99%	99.9%
Athletes/endurance‐trained individuals (*n* = 10,000)	100 μg at 8/16 h	Directly after inhalation	1.4	3.6	9.6	11.5	15.8	0.1	0.9	4.6	6.1	10.1
		30 min post‐dose	0.7	2.0	5.6	6.8	10.0	0.4	1.7	7.0	9.0	14.8
	100 μg at 8/20 h	Directly after inhalation	1.4	3.6	9.4	11.3	15.6	0.0	0.4	3.0	4.3	8.3
		30 min post‐dose	0.6	1.9	5.3	6.5	9.0	0.3	1.2	5.5	7.3	11.9
	200 μg at 8 h	Directly after inhalation	2.6	6.9	18.4	22.1	34.1	0.0	0.1	1.1	1.8	3.7
		30 min post‐dose	1.2	3.5	10.0	12.3	17.8	0.4	1.7	6.6	8.5	13.8
	200 μg at 8/16 h	Directly after inhalation	2.8	7.3	19.2	23.0	31.6	0.3	1.7	9.2	12.2	20.3
		30 min post‐dose	1.4	3.9	11.2	13.7	20.0	0.8	3.3	13.9	18.1	29.5
	200 μg at 8/20 h	Directly after inhalation	2.7	7.2	19.2	23.2	34.7	0.1	0.8	6.0	8.3	15.2
		30 min post‐dose	1.3	3.8	10.8	13.2	20.4	0.6	2.5	10.9	13.9	25.1
Healthy participants (*n* = 10,000)	100 μg at 8/16 h	Directly after inhalation	0.5	1.3	3.5	4.2	5.9	0.3	1.6	8.2	10.8	17.8
		30 min post‐dose	0.3	0.8	2.2	2.7	4.0	0.6	2.5	10.7	13.9	22.3
	100 μg at 8/20 h	Directly after inhalation	0.5	1.3	3.4	4.1	5.7	0.1	1.0	6.4	9.1	16.2
		30 min post‐dose	0.2	0.7	2.1	2.5	3.5	0.4	1.9	8.8	11.9	19.4
	200 μg at 8 h	Directly after inhalation	0.9	2.4	6.4	7.7	11.9	0.0	0.3	3.4	5.1	9.5
		30 min post‐dose	0.5	1.3	3.7	4.5	6.6	0.5	2.1	8.7	11.3	19.7
	200 μg at 8/16 h	Directly after inhalation	1.0	2.7	7.1	8.4	11.7	0.6	3.3	16.3	21.5	35.6
		30 min post‐dose	0.5	1.6	4.5	5.4	7.9	1.1	4.9	21.3	27.7	44.7
	200 μg at 8/20 h	Directly after inhalation	1.0	2.6	6.9	8.4	12.4	0.3	2.1	12.8	17.1	32.1
		30 min post‐dose	0.5	1.5	4.3	5.3	7.9	0.8	3.8	17.7	22.8	41.6

*Note:* For the regimen in which salmeterol is administered twice a day, the predicted concentrations displayed correspond to those following the second dose. Permitted and prohibited regimens are separated by a horizontal thick line.

## 4 Discussion

Our popPK model allowed the simultaneous description of plasma and urine concentrations of salmeterol and its metabolite α‐hydroxysalmeterol. A two‐compartment model with an intravenous‐like absorption was found to best depict plasma salmeterol PK, consistent with previous analyses [21]. Our findings, in accordance with previous observations [6, 7], show that maximal salmeterol plasma concentrations are observed within the first 15 min after inhalation. The contribution of the gastrointestinal absorption of the swallowed drug may occur after 45–90 min [6]. Therefore, a parallel absorption was tested but was not retained to better estimate the remaining parameters and variability components. Regarding the observed differences in covariate effects between athletes and other participants, the higher apparent drug clearance in athletes could potentially be attributed to training‐induced changes in hepatic blood flow or minor alterations in protein binding, for instance. In addition, the substantial apparent volumes of distribution indicate extensive distribution throughout the body owing to the high lipophilicity of salmeterol. Our model allowed the estimation of average t_1/2_ for plasma salmeterol of 0.13 and 5 h, corresponding to the initial decline due to diffusion into the peripheral compartment and the onset of elimination, and a final decline due to elimination after reaching equilibrium, respectively. Final model parameters are all well estimated, except for the IIV of 16% on $V_{1}/F$ (RSE 54%). Although this variability is relatively low and moderately well estimated, it was decided to retain it based on the diagnostic plots evaluation, its coherent bootstrap estimation, in addition to the observed reduction in the OFV (ΔOFV = −37, *p* < 0.0001).

Our popPK model was developed using a single dataset including plasma concentration data, which may limit the robustness of the predictions of the model for other populations. However, the model was informed by several datasets containing urine concentration data, which provided additional insight into drug disposition. Although no external validation could be performed, internal validation techniques such as bootstrapping and pcVPCs were used to assess the reliability of the model. The concentrations of α‐hydroxysalmeterol in plasma were only available in the study by Jessen et al. [11], which included endurance‐trained individuals. Due to the low concentrations measured, it appears that the data lack granularity, probably hampering the estimation of the IIVs on the clearance of α‐hydroxysalmeterol (CL_α_/F) and its excretion in urine (k_35_). However, these data provided valuable information for describing urine concentrations of α‐hydroxysalmeterol. Constraining $V_{1}$ = $V_{3}$ and $V_{4}$ = $V_{5}$ was required for model identifiability; while this does not affect the simulated plasma or urine concentrations, it limits the interpretation of the absolute fractions eliminated via specific routes. On another note, the average hourly urine production was estimated at 79 mL/h (with 73% IIV for USG‐uncorrected urine concentrations), which is in fair accordance with the typical daily urine volume of 1–2 L produced. However, the model may underestimate the variability in urine production. Our approach assumes a constant urine production rate, which ignores the intrinsic fluctuations of physiological micturition. Dehydration and subsequent rehydration, particularly in the context of prolonged strenuous physical activity during professional competition, could further increase this variability. The observed covariate effects may be confounded by unaccounted‐for variability in hydration levels and urine volumes in samples lacking USG correction. Due to the limited availability of relevant covariates, such as the individual demographic characteristics or physical activity, we were not able to further explain some of the IIV. Finally, the dataset's limited size and heterogeneity precluded external validation, as splitting it into separate training and validation sets would have reduced the statistical power needed for reliable parameter estimation and covariate identification. Additional studies including diverse populations remain therefore suitable to ascertain whether our findings can be generalized. Specifically, research focusing on athletes with asthma is required to quantify the combined, potentially compensatory impact of the disease state and athlete status on CL/F.

A key finding of our study was that salmeterol urine concentrations after therapeutic inhalation up to 200 μg once daily are not expected to be reported under the current MRL, which is designed to minimize the likelihood of reporting adverse analytical findings from athletes adhering to the permitted therapeutic regimen. In fact, our model‐based simulations also suggest a very low probability of exceeding the MRL after repeated prohibited inhaled doses up to 400 μg per day, corresponding to twice the allowed regimen. Higher doses, more susceptible to cause urine concentrations above the current MRL, could lead to toxicities such as skeletal muscle tremors, headache, nausea, fatigue, or heart palpitations [7]. In particular, at high inhaled doses, β_2_‐agonists enhance intense exercise performance and muscle strength as well as induce muscle hypertrophic effects. However, they also induce long‐term detrimental effects on aerobic performance and impair oxidative capacity [4].

Our model‐based simulations suggest that the current rule (i.e., dosing limit of 200 μg in any 24 h period via inhaled administration of salmeterol) could be refined to improve the likelihood of reporting suspected cases of supratherapeutic use. Indeed, as no drug accumulation is expected, it may be difficult to distinguish between a permitted regimen of 200 μg salmeterol once daily and a prohibited dose of 200 μg salmeterol twice daily. An adjustment to the current rule to set a dose limit of 100 μg per 12‐h period for inhaled salmeterol, in line with therapeutic recommendations [7], could already have significant practical relevance. Caution is warranted regarding the reported 99.9% probability in our results, as its precision is inherently poor and highly sensitive to unverifiable assumptions in the extreme tails of the Gaussian distribution. Only the order of magnitude can reasonably be retained. The a priori predictions generated by our popPK model could also enable the calculation of the individual's PK parameters through maximum likelihood estimation using a Bayesian approach [27]. Repeated measurements collected from an athlete could reduce the prediction uncertainty of their individual PK profile, thereby helping to better distinguish therapeutic use from the risk of salmeterol misuse. Lastly, given the higher overall urine concentrations of α‐hydroxysalmeterol compared to salmeterol, it may be more feasible to develop reliable analytical methods for this compound. In addition, as illustrated in simulations, the concentrations of α‐hydroxysalmeterol in urine appear to be less variable over time, thus offering more flexibility in the scheduling of urine sampling. A different approach could therefore use an MRL or Threshold on the α‐hydroxysalmeterol metabolite, assuming that appropriate reference materials and a deuterated internal standard would be available to the anti‐doping laboratories. However, it should be noted that such an approach would suffer, to some extent, from the same limitation as for salmeterol, particularly with regard to limited metabolite accumulation.

In conclusion, our popPK model appears able to effectively describe the concentrations of salmeterol and its metabolite, α‐hydroxysalmeterol, in both plasma and urine. The present analysis represents a first step towards refining the MRL‐approach for the assessment of salmeterol use in athletes. Further studies are warranted to collect more data across diverse populations to extend the generalizability of our findings.

## Author Contributions

P.T. wrote the manuscript. P.T., A.D., T.B., F.R.G., I.M., O.R., and M.G. designed the research. M.H., M.P., and K.D. performed the research. P.T. and M.G. analyzed the data.

## Funding

This work was supported by funding from the World Anti‐Doping Agency.

## Conflicts of Interest

The authors declare no conflicts of interest.

## Supporting information


**Data S1:** Supporting information.

## Data Availability

No additional data is available.
