# Supplementary material for: Pharmacometric‐Based Evaluation of Salmeterol and Its Metabolite α‐Hydroxysalmeterol in Plasma and Urine: Practical Implications for Doping Control
Source: CPT Pharmacometrics Syst Pharmacol. 2026 Jan 16;15(2):e70187. doi: 10.1002/psp4.70187 (PMC12823318; doi:10.1002/psp4.70187)
Supplement: Supplementary file 1 — Data S1: Supporting information. [file PSP4-15-e70187-s001.docx]

**Supplementary Material**

[Figure S1: Flow diagram of literature search and study selection. 2](#_Toc214624639)

[Data formatting description 3](#_Toc214624640)

[Figure S2: Goodness-of-fit diagnostic plots of the final model. 6](#_Toc214624641)

[Figure S3: Comparison of the IIV distribution between the base model and the covariate model. 7](#_Toc214624642)

[Figure S4: Prediction-corrected visual predictive checks of the final popPK model. 8](#_Toc214624643)

[Figure S5: Simulated percentiles of salmeterol and α-hydroxysalmeterol in plasma and in urine. 9](#_Toc214624644)

[Table S1: Predicted α-hydroxysalmeterol urine concentrations for the different salmeterol regimens. 10](#_Toc214624645)

[NONMEM script 11](#_Toc214624646)

[References 14](#_Toc214624647)


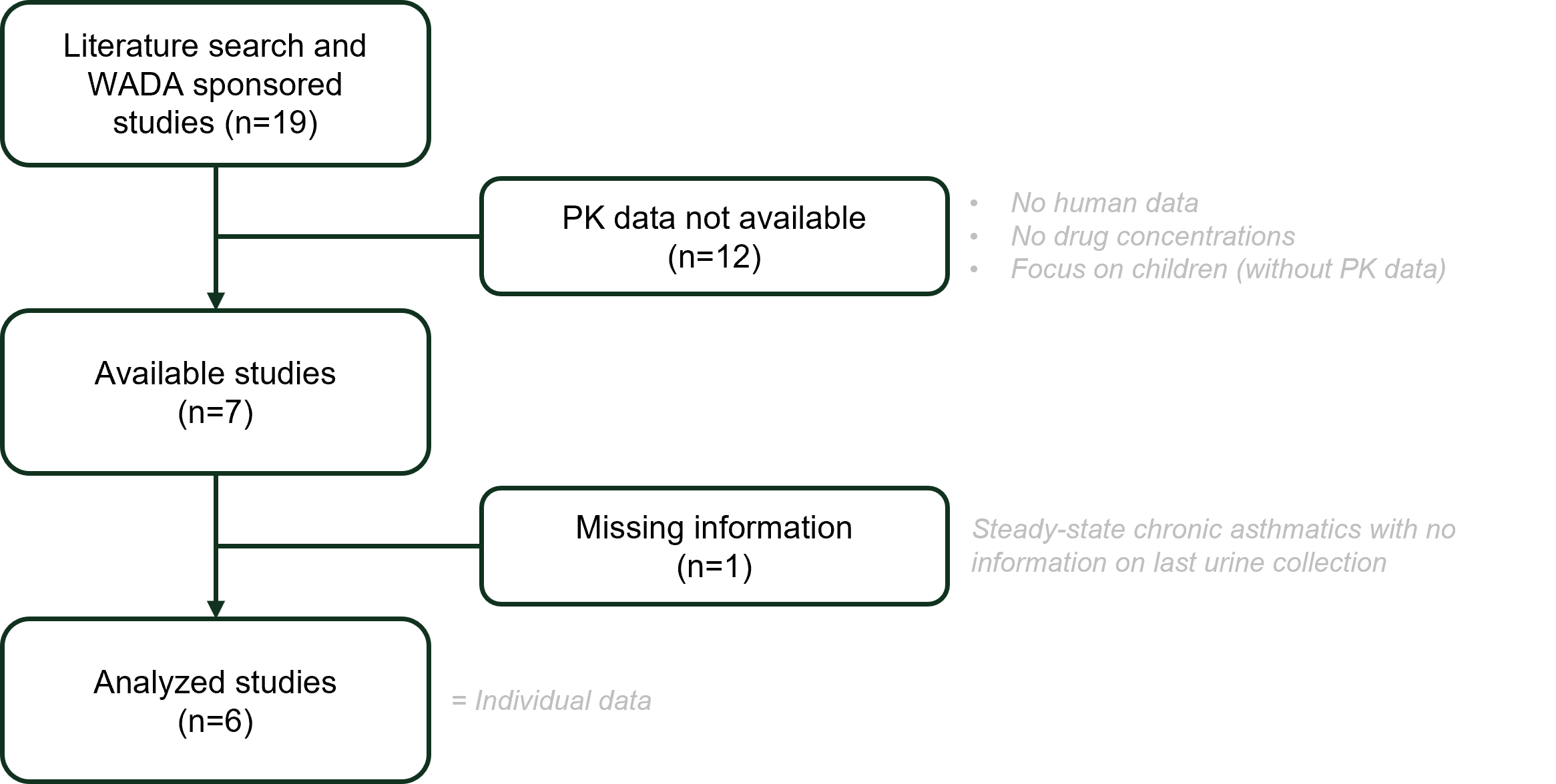


# Figure S1: Flow diagram of literature search and study selection.

## Data formatting description

Three distinct scenarios were necessary to format urine data for NONMEM analysis:

1. The ideal situation with recorded urine volume collected at each sampling time (two studies^1,2^).
   In this case, the NONMEM dataset contains urine compartments opening (i.e., CMT>0, MDV=1, EVID=2) and closing (i.e., CMT<0, MDV=0, EVID=2) with urine volumes explicitly assigned as follows:

| **ID** | **TIME** | **AMT** | **DV** | **CMT** | **EVID** | **MDV** | **UVOL** | **RATE** |
| --- | --- | --- | --- | --- | --- | --- | --- | --- |
| 22 | 0 | 962.52 | . | 1 | 1 | 1 | . | 0 |
| 22 | 0 | . | . | 4 | 2 | 1 | . | 0 |
| 22 | 0 | . | . | 5 | 2 | 1 | . | 0 |
| 22 | 2 | . | 2.33 | -4 | 0 | 0 | 0.29 | 0 |
| 22 | 2 | . | 8.67 | -5 | 0 | 0 | 0.29 | 0 |
| 22 | 2 | . | . | 4 | 2 | 1 | . | 0 |
| 22 | 2 | . | . | 5 | 2 | 1 | . | 0 |

In this first example, urine collection is assumed just before the dosing (i.e., compartments are opened at the same time of dosing, representing bladder filling), followed by urine collection at 2 hours post-dose (i.e., negative compartments representing bladder voiding together with the specified volume of urine collected (UVOL, in L)). The urine compartments are turned back on to start a new urine collection. Salmeterol urine data are fitted with the code as follows:

…

IF (CMT.EQ.-4.AND.EVID.NE.2) THEN *; Example for compartment -4 with proportional error*

IPRED = A(4)/UVOL

Y = IPRED*EXP(ERR(1))

ENDIF

…

The same code was implemented to fit α-hydroxysalmeterol data with the corresponding compartments and amount, A(5).

Note: The condition “EVID.NE.2” specifies that only lines with recorded observations should be fitted.

1. The most frequent situation where urine volumes were collected without documentation of the time of collection (three studies^3-5^).

In this case, a separate urine compartment was defined and added in the dataset to approximate physiologic micturition assuming constant urine production (UR_PROD, i.e., compartment 6 describing the urine production per hour), as follows:

| **ID** | **TIME** | **AMT** | **DV** | **CMT** | **EVID** | **MDV** | **UVOL** | **RATE** |
| --- | --- | --- | --- | --- | --- | --- | --- | --- |
| 1 | 0 | 240.63 | . | 1 | 1 | 1 | . | 0 |
| 1 | 0 | . | . | 4 | 2 | 1 | . | 0 |
| 1 | 0 | . | . | 5 | 2 | 1 | . | 0 |
| 1 | 0 | . | . | 6 | 2 | 1 | . | 0 |
| 1 | 4 | . | 1.35 | 4 | 0 | 0 | . | 0 |
| 1 | 4 | . | . | -4 | 2 | 1 | . | 0 |
| 1 | 4 | . | 6.89 | 5 | 0 | 0 | . | 0 |
| 1 | 4 | . | . | -5 | 2 | 1 | . | 0 |
| 1 | 4 | . | . | -6 | 2 | 1 | . | 0 |
| 1 | 4 | . | . | 4 | 2 | 1 | . | 0 |
| 1 | 4 | . | . | 5 | 2 | 1 | . | 0 |
| 1 | 4 | . | . | 6 | 2 | 1 | . | 0 |

Such data formatting assumes that urine production begins at the same time as dosing. Then, at the time of urine collection, observations and bladder voiding are specified separately, but it must be ensured that the observation precedes the bladder voiding. The urine compartments are subsequently turned back on for the start of a new urine collection. Salmeterol urine data are fitted as follows dividing the drug amounts by the volume produced in the corresponding period:

…

IF (CMT.EQ.4.AND.EVID.NE.2) THEN ; *Example for compartment 4 with proportional error*

IPRED = A(4)/A(6)

Y = IPRED*EXP(ERR(1))

ENDIF

…

The same code was implemented to fit α-hydroxysalmeterol data with the corresponding compartments and amount, A(5).

1. Lastly, the scenario of the study by Petrou *et al.* (unpublished, see Table 1) where the time of the last urine collection before dosing was recorded, but without information on the collected volume. Therefore, a similar approach to scenario 2 was implemented using the additional compartment for urine production at the time of last urine collection that occurred before salmeterol administration. Urine data were formatted as follows:

| **ID** | **TIME** | **AMT** | **DV** | **CMT** | **EVID** | **UVOL** | **RATE** |
| --- | --- | --- | --- | --- | --- | --- | --- |
| 83 | 0 | . | . | 6 | 2 | . | 0 |
| 83 | 0.7 | 240.63 | . | 1 | 1 | . | 0 |
| 83 | 0.7 | . | . | 4 | 2 | . | 0 |
| 83 | 0.7 | . | . | 5 | 2 | . | 0 |
| 83 | 2.72 | . | 0.21 | 4 | 0 | . | 0 |
| 83 | 2.72 | . | . | -4 | 2 | . | 0 |
| 83 | 2.72 | . | 2.96 | 5 | 0 | . | 0 |
| 83 | 2.72 | . | . | -5 | 2 | . | 0 |
| 83 | 2.72 | . | . | -6 | 2 | . | 0 |
| 83 | 2.72 | . | . | 4 | 2 | . | 0 |
| 83 | 2.72 | . | . | 5 | 2 | . | 0 |
| 83 | 2.72 | . | . | 6 | 2 | . | 0 |

The same code of scenario 2 was used to fit salmeterol and α-hydroxysalmeterol data.

For all scenarios, the option INITIALOFF NODOSE in the $MODEL section needs to be specified to allow for negative compartments to be implemented (see NONMEM^®^ script).


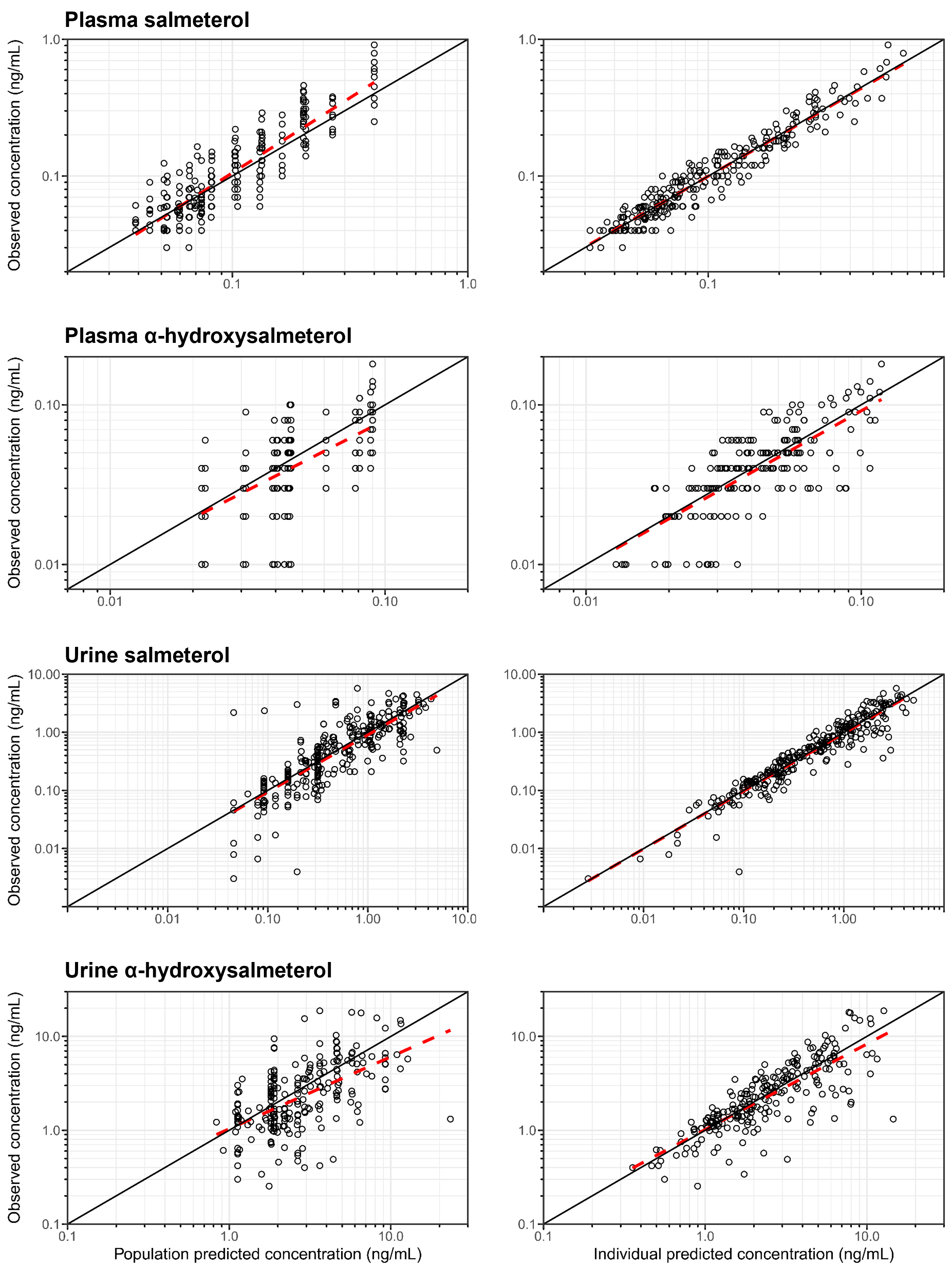


# Figure S2: Goodness-of-fit diagnostic plots of the final model.

Note that the plasma concentrations of α-hydroxysalmeterol lacks in granularity due to the very low concentrations measured (pg/mL range), resulting in a horizontal arrangement of the data along the line of identity.





# Figure S3: Comparison of the IIV distribution between the base model and the covariate model.

$\eta_{i}$: inter-individual variability.


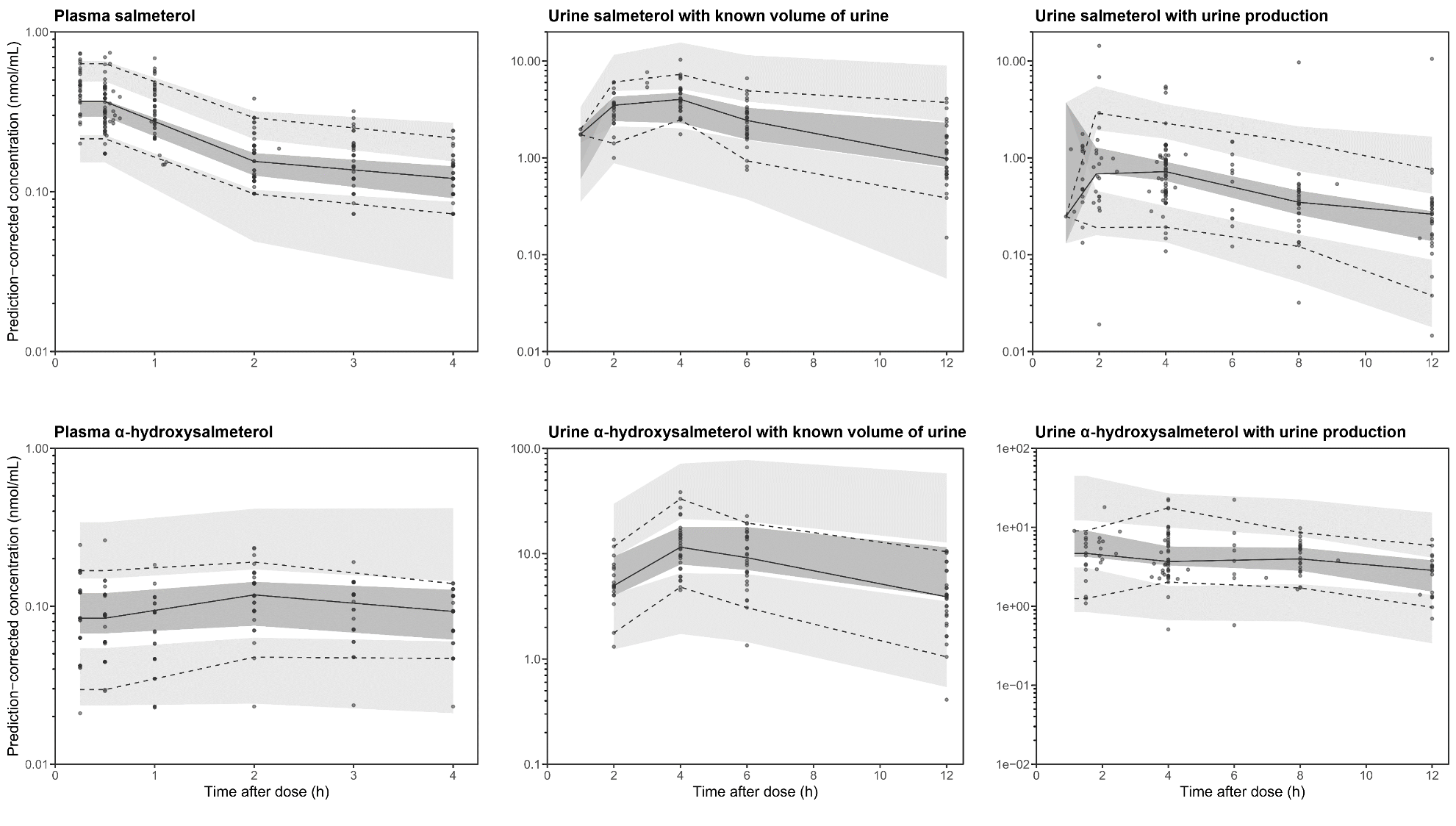


# Figure S4: Prediction-corrected visual predictive checks of the final popPK model.

Open circles represent the prediction-corrected observed plasma concentrations. Solid and dashed lines represent the median and 90% prediction intervals (PI_90%_) of the prediction-corrected observed data, respectively. Shaded surfaces represent the model-predicted 90% confidence intervals of the prediction-corrected simulated median and PI_90%_.


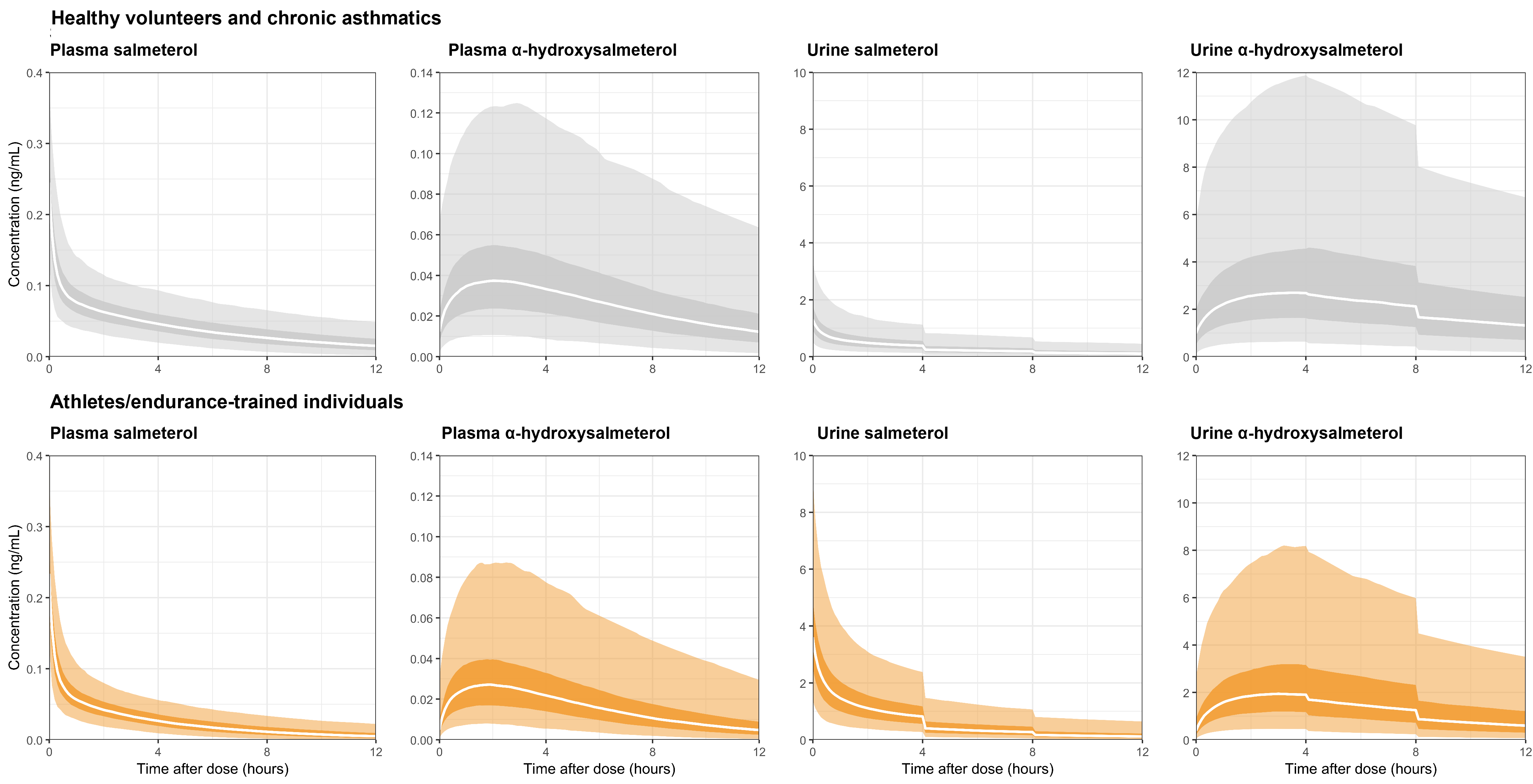


# Figure S5: Simulated percentiles of salmeterol and α-hydroxysalmeterol in plasma and in urine.

An authorized dose of 100 µg salmeterol every 12h for one week with bladder voiding every 4 hours was simulated (n=1000). Urine concentrations represent urine specific gravity-corrected concentrations. The solid white lines represent the median (50% percentile), while the dark surfaces encompass the 50% prediction intervals, and the light surfaces the 95% prediction intervals. Each point of the simulated urine PK profile represents the concentration that would be obtained if a urine sample were collected at that time point.

# Table S1: Predicted α-hydroxysalmeterol urine concentrations for the different salmeterol regimens.

|  | | **Athletes/endurance-trained individuals (n=10,000)**  *α-hydroxysalmeterol concentration (ng/mL)* | | | | | **Healthy volunteers (n=10,000)**  *α-hydroxysalmeterol concentration (ng/mL)* | | | | |
| --- | --- | --- | --- | --- | --- | --- | --- | --- | --- | --- | --- |
|  |  | **2.5%** | **50%** | **97.5%** | **99%** | **99.9%** | **2.5%** | **50%** | **97.5%** | **99%** | **99.9%** |
| **100 µg at 8-16h** | 2h post-dose | 0.5 | 2.2 | 9.1 | 12.0 | 19.9 | 0.7 | 3.1 | 13.1 | 17.0 | 27.7 |
|  | 3h post-dose | 0.5 | 2.3 | 9.4 | 12.6 | 20.8 | 0.7 | 3.2 | 13.5 | 17.8 | 29.5 |
|  | 4h post-dose | 0.5 | 2.3 | 9.3 | 12.5 | 20.8 | 0.7 | 3.2 | 13.5 | 18.0 | 29.8 |
| **100 µg at 8-20h** | 2h post-dose | 0.4 | 1.9 | 7.8 | 10.5 | 16.6 | 0.6 | 2.6 | 11.3 | 15.6 | 25.7 |
|  | 3h post-dose | 0.5 | 2.0 | 8.2 | 11.3 | 17.3 | 0.6 | 2.7 | 11.9 | 16.5 | 26.4 |
|  | 4h post-dose | 0.5 | 2.0 | 8.3 | 11.5 | 17.5 | 0.6 | 2.8 | 12.2 | 16.8 | 26.2 |
| **200 µg at 8h** | 2h post-dose | 0.8 | 3.0 | 11.7 | 15.1 | 26.1 | 0.9 | 3.7 | 14.5 | 18.7 | 31.3 |
|  | 3h post-dose | 0.8 | 3.3 | 12.9 | 16.9 | 28.3 | 1.0 | 4.0 | 15.9 | 20.8 | 34.5 |
|  | 4h post-dose | 0.8 | 3.3 | 13.1 | 17.5 | 28.5 | 1.0 | 4.2 | 16.7 | 22.1 | 36.9 |
| **200 µg at 8-16h** | 2h post-dose | 1.0 | 4.5 | 18.3 | 24.0 | 39.9 | 1.4 | 6.3 | 26.2 | 33.9 | 55.5 |
|  | 3h post-dose | 1.0 | 4.6 | 18.8 | 25.2 | 41.6 | 1.4 | 6.5 | 27.0 | 35.5 | 59.1 |
|  | 4h post-dose | 1.0 | 4.5 | 18.7 | 25.0 | 41.5 | 1.4 | 6.5 | 27.1 | 35.9 | 59.7 |
| **200 µg at 8-20h** | 2h post-dose | 0.9 | 3.8 | 15.5 | 20.1 | 34.5 | 1.2 | 5.3 | 22.8 | 29.2 | 52.5 |
|  | 3h post-dose | 0.9 | 3.9 | 16.3 | 21.1 | 36.8 | 1.3 | 5.6 | 23.8 | 31.0 | 55.0 |
|  | 4h post-dose | 0.9 | 3.9 | 16.5 | 21.4 | 36.6 | 1.2 | 5.6 | 24.2 | 31.4 | 56.3 |

Note: For the regimen in which salmeterol is administered twice a day, the predicted concentrations displayed correspond to those following the second dose.

Permitted and prohibited regimens are separated by a horizontal thick line.

## NONMEM script

$SIZES LVR=50 LNP4=10000

$PROBLEM PK

$INPUT ID TIME AMT DV CMT EVID OCC MDV SS II TYPE DEVICE STUDY SID UVOL USG BQL L2 RATE

$DATA Salmeterol_M1_L2.csv IGNORE=#

$SUBROUTINES ADVAN13 TOL=6

$MODEL ;------------------------------------------------------------------------------------------------------------------------------------------------------------

NCOMP=6

COMP = (PARENT_PLASMA, DEFDOSE)

COMP = (PLASMA_PERIPH)

COMP = (METAB_PLASMA)

COMP = (PARENT_URINE INITIALOFF NODOSE)

COMP = (METAB_URINE INITIALOFF NODOSE)

COMP = (UR_PROD)

$PK ;-------------------------------------------------------------------------------------------------------------------------------------------------------------------

IF(AMT.GT.0) THEN

TDOS=TIME

TAD=0.0

ENDIF

IF (AMT.EQ.0) TAD=TIME-TDOS

; --------------------------------------------

ATHLETES = 0

IF (TYPE.EQ.3) ATHLETES = 1

; --------------------------------------------

TVV1 = THETA(1)

V1 = TVV1 * EXP(ETA(1))

V3 = V1

TVQ = THETA(2)

Q = TVQ * EXP(ETA(2))

TVV2 = THETA(3)

V2 = TVV2 * EXP(ETA(3))

TVCLP = THETA(4)*THETA(10)**ATHLETES

CLP = TVCLP * EXP(ETA(4))

TVCLM = THETA(5)

CLM = TVCLM * EXP(ETA(5))

TVK13 = THETA(6)

K13 = TVK13 * EXP(ETA(6))

TVK14 = THETA(7)*THETA(11)**ATHLETES

K14 = TVK14 * EXP(ETA(7))

TVK35 = THETA(8)

K35 = TVK35 * EXP(ETA(8))

TVUR_PROD = THETA(9)

IF(USG.EQ.0) THEN

UR_PROD = TVUR_PROD * EXP(ETA(9))

ENDIF

IF(USG.EQ.1) THEN

UR_PROD = TVUR_PROD * EXP(ETA(10))

ENDIF

K10 = CLP/V1 - K13 - K14

K30 = CLM/V3 - K35

K21 = Q/V2

K12 = Q/V1

S1 = V1

S3 = S1

$DES ;----------------------------------------------------------------------------------------------------------------------------------------------------------------

DADT(1) = K21*A(2) - K12*A(1) - K13*A(1) - K14*A(1) - K10*A(1)

DADT(2) = K12*A(1) - K21*A(2)

DADT(3) = K13*A(1) - K35*A(3) - K30*A(3)

DADT(4) = K14*A(1)

DADT(5) = K35*A(3)

DADT(6) = UR_PROD

$ERROR ;------------------------------------------------------------------------------------------------------------------------------------------------------------

; Plasma ------------------------------------------------------------------------------------------------------------------------

IF (CMT.EQ.1) THEN ; Jessen *et al.* ^1^, Hostrup *et al.* ^5^, Petrou *et al.* (unpublished)

IPRED = A(1)/S1

Y = IPRED*EXP(ERR(1))

ENDIF

IF (CMT.EQ.3) THEN ; Jessen *et al.* ^1^

IPRED = A(3)/S3

Y = IPRED*EXP(ERR(2))

ENDIF

; Urine salmeterol ------------------------------------------------------------------------------------------------------------

IF (CMT.EQ.4.AND.SID.EQ.1.AND.EVID.NE.2) THEN ; Jacobson *et al.* ^3^

IPRED = A(4)/A(6)

Y = IPRED*EXP(ERR(3))

ENDIF

IF (CMT.EQ.-4.AND.SID.EQ.2.AND.EVID.NE.2) THEN ; Jessen *et al.* ^1^ (known urine volumes)

IPRED = A(4)/UVOL

Y = IPRED*EXP(ERR(5))

ENDIF

IF (CMT.EQ.4.AND.SID.EQ.4.AND.EVID.NE.2) THEN ; Jacobson *et al.* ^4^

IPRED = A(4)/A(6)

Y = IPRED*EXP(ERR(7))

ENDIF

IF (CMT.EQ.4.AND.SID.EQ.5.AND.EVID.NE.2) THEN ; Hostrup *et al.* ^5^

IPRED = A(4)/A(6)

Y = IPRED*EXP(ERR(3))

ENDIF

IF (CMT.EQ.-4.AND.SID.EQ.6.AND.EVID.NE.2) THEN ; Deventer *et al.* ^2^ (known urine volumes)

IPRED = A(4)/UVOL

Y = IPRED*EXP(ERR(3))

ENDIF

IF (CMT.EQ.4.AND.SID.EQ.7.AND.EVID.NE.2) THEN ; Petrou *et al.* (unpublished)

IPRED = A(4)/A(6)

Y = IPRED*EXP(ERR(5))

ENDIF

; Urine OH-salmeterol -------------------------------------------------------------------------------------------------------

IF (CMT.EQ.5.AND.SID.EQ.1.AND.EVID.NE.2) THEN ; Jacobson *et al.* ^3^

IPRED = A(5)/A(6)

Y = IPRED*EXP(ERR(4))

ENDIF

IF (CMT.EQ.-5.AND.SID.EQ.2.AND.EVID.NE.2) THEN ; Jessen *et al.* ^1^ (known urine volumes)

IPRED = A(5)/UVOL

Y = IPRED*EXP(ERR(6))

ENDIF

IF (CMT.EQ.5.AND.SID.EQ.5.AND.EVID.NE.2) THEN ; Hostrup *et al.* ^5^

IPRED = A(5)/A(6)

Y = IPRED*EXP(ERR(4))

ENDIF

IF (CMT.EQ.5.AND.SID.EQ.7.AND.EVID.NE.2) THEN ; Petrou *et al.* (unpublished)

IPRED = A(5)/A(6)

Y = IPRED*EXP(ERR(4))

ENDIF

$THETA ; *Final estimates* ---------------------------------------------------------------------------------------------------------------------------------------

446 ; V1/F

1490 ; Q/F

871 ; V2/F

193 ; CLP/F = Salmeterol clearance

233 ; CLM/F = α-hydroxysalmeterol clearance

0.3 ; K13

0.000943 ; K14

0.0147 ; K35

0.079 ; UR_PROD

1.63 ; ATH-CLP

2.91 ; ATH-K14

$OMEGA ; *Final estimates* --------------------------------------------------------------------------------------------------------------------------------------

0.0262 ; IIV V1

0.554 ; IIV Q

0.171 ; IIV V2

0.103 ; IIV CLP = Salmeterol IIV

0 FIX ; IIV CLM = α-hydroxysalmeterol IIV

0.157 ; IIV K13

0.081 ; IIV K14

0 FIX ; IIV K35

0.429 ; IIV UR_PROD not corrected

0 FIX ; IIV UR_PROD corrected

$SIGMA BLOCK(2) ; *Final estimates* -------------------------------------------------------------------------------------------------------------------------

0.0461 ; Prop - Plasma salmeterol (Jessen *et al.* ^1^, Hostrup *et al.* ^5^, Petrou *et al.*)

0.0422 0.145 ; Prop - Plasma OH salmeterol, (Jessen *et al.* ^1^)

$SIGMA

0.0847 ; Prop - Urine salmeterol (Jacobson *et al.* ^3^, Hostrup *et al.* ^5^, Deventer *et al.* ^2^)

0.144 ; Prop - Urine OH-salmeterol (Jacobson *et al.* ^3^, Hostrup *et al.* ^5^, Petrou *et al.*)

$SIGMA BLOCK(2)

0.166 ; Prop - Urine salmeterol (Jessen *et al.* ^1^, Petrou *et al.*)

0.127 0.264 ; Prop - Urine OH-salmeteroel (Jessen *et al.* ^1^)

$SIGMA

0.322 ; Prop - Urine salmeterol (Jacobson *et al.* ^4^)

;-------------------------------------------------------------------------------------------------------------------------------------------------------------------------

$EST METHOD=1 INTER MAXEVAL=9999 NOABORT PRINT=5 SIGL=6 NSIG=2

$COV MATRIX=S

## References

1. Jessen S, Becker V, Rzeppa S, et al. Pharmacokinetics of salmeterol and its main metabolite α-hydroxysalmeterol after acute and chronic dry powder inhalation in exercising endurance-trained men: Implications for doping control. *Drug Testing and Analysis* 2021; **13**(4): 747-61.

2. Deventer K, Pozo OJ, Delbeke FT, Van Eenoo P. Quantitative detection of inhaled salmeterol in human urine and relevance to doping control analysis. *Ther Drug Monit* 2011; **33**(5): 627-31.

3. Jacobson GA, Hostrup M. The salmeterol anomaly and the need for a urine threshold. *Drug Testing and Analysis* 2022; **14**(6): 997-1003.

4. Jacobson GA, Hostrup M, Narkowicz CK, Nichols DS, Haydn Walters E. Enantioselective disposition of (R)-salmeterol and (S)-salmeterol in urine following inhaled dosing and application to doping control. *Drug Test Anal* 2017; **9**(8): 1262-6.

5. Hostrup M, Kalsen A, Elers J, et al. Urine concentrations of inhaled salmeterol and its metabolite alpha-hydroxysalmeterol in asthmatic and non-asthmatic subjects. *Journal of Sports Medicine & Doping Studies* 2012; **2**(2).
